# Acetylcholine and spinal locomotor networks: The insider

**DOI:** 10.14814/phy2.14736

**Published:** 2021-02-01

**Authors:** Théo Mille, Camille Quilgars, Jean‐René Cazalets, Sandrine S. Bertrand

**Affiliations:** ^1^ Université de Bordeaux CNRS UMR 5287 INCIA Bordeaux France

## Abstract

This article aims to review studies that have investigated the role of neurons that use the transmitter acetylcholine (ACh) in controlling the operation of locomotor neural networks within the spinal cord. This cholinergic system has the particularity of being completely intraspinal. We describe the different effects exerted by spinal cholinergic neurons on locomotor circuitry by the pharmacological activation or blockade of this propriospinal system, as well as describing its different cellular and subcellular targets. Through the activation of one ionotropic receptor, the nicotinic receptor, and five metabotropic receptors, the M1 to M5 muscarinic receptors, the cholinergic system exerts a powerful control both on synaptic transmission and locomotor network neuron excitability. Although tremendous advances have been made in our understanding of the spinal cholinergic system's involvement in the physiology and pathophysiology of locomotor networks, gaps still remain, including the precise role of the different subtypes of cholinergic neurons as well as their pre‐ and postsynaptic partners. Improving our knowledge of the propriospinal cholinergic system is of major relevance to finding new cellular targets and therapeutics in countering the debilitating effects of neurodegenerative diseases and restoring motor functions after spinal cord injury.

## Graphical abstract

This article aims to review studies that have investigated the role of neurons that use the transmitter acetylcholine in controlling the operation of locomotor neural networks within the spinal cord.

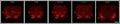

## 1 INTRODUCTION

In the spinal cord, to generate locomotor activity, neurons are organized into highly specialized networks called central pattern generators (CPGs), which are endowed with the ability to generate in an autonomous manner both the rhythm itself and the pattern (the precise sequence of muscular activation) of locomotor activity. The motoneurons (MNs) innervating the various effector muscles receive this central command and convert it into contractions through the neuromuscular junctions.

To enable the continual adaptation of the motor command to external and internal demands, both CPG neurons and MNs are targeted by sensory, intraspinal, and supraspinal synaptic inputs that are extremely diverse in terms of neurochemical phenotype. Classically, a distinction has been made between neurotransmitters that sustain fast chemical transmission between neurons through the activation of ion channel receptors: glutamate, GABA and glycine, and neuromodulators: monoamines, acetylcholine, peptides… whose binding to metabotropic receptors activates intracellular second messenger cascades leading to delayed and prolonged postsynaptic responses. It is now well acknowledged that this distinction is not so clear, with many neuroactive compounds being capable of activating both ionotropic and metabotropic receptors simultaneously. An impressive amount of work has been done to assess the role of the different neurotransmitter/neuromodulatory systems in the physiology and physiopathology of spinal locomotor networks (for reviews see for examples: Garcia‐Campmany et al., 2010; Jordan et al., 2008; Katz, 1995; Kiehn, 2016; Miles & Sillar, 2011; Rekling et al., 2000; Sharples et al., 2014).

The goal of this article was to review the cholinergic system that controls spinal locomotor networks, which is a neuromodulatory system that has the particularity of being completely intraspinal—all cholinergic neurons that connect to spinal motor networks reside within the spinal cord. This is in sharp contrast to the monoaminergic system, for example, whose neurons are located mostly in supraspinal areas and exert a major neuromodulatory control of spinal circuitry through descending pathways.

## 2 CHOLINERGIC SYSTEMS

Acetylcholine (ACh), the first chemical transmitter shown to be released by neurons, is produced from acetyl‐CoA and choline by the enzyme choline acetyltransferase (ChAT) and is loaded into synaptic vesicles by the vesicular acetylcholine transporter (VAChT). One characteristic of ACh is its very fast degradation after release by the enzyme acetylcholinesterase (AChE), thereby limiting the transmitter's accumulation in the synaptic cleft. Cholinergic neurons are spread in diverse nuclei throughout the central nervous system (CNS) such as the pedunculopontine and laterodorsal tegmental areas, medial habenula, basal forebrain, and the spinal cord. Acetylcholine is implied in multiple nervous functions such as arousal, sleep cycle regulation, learning and memory, sensory integration, and motor control (for reviews see for examples: Haam & Yakel, 2017; Miles & Sillar, 2011; Picciotto et al., 2012; Thiele & Bellgrove, 2018; Umana et al., 2017). Alterations in cholinergic systems have been linked to numerous nervous disorders including Alzheimer and Parkinson's diseases, addiction, biological rhythm perturbation, and depression (for reviews see for examples: Dulawa & Janowsky, 2018; Ferreira‐Vieira et al., 2016; Picciotto et al., 2012; Ztaou & Amalric, 2019).

The physiological actions of ACh are mediated by two functionally different types of receptors: one ionotropic receptor, the nicotinic receptor (nAChR) permeant to cations (Zoli et al., 2014) and five types of metabotropic receptors, the muscarinic receptors (mAChRs; M1 to M5). The latter trigger opposing responses in target neurons according to the G‐protein subtype they activate, with M1, M3, and M5 coupled to G_q/11_ and M2–M4 to G_i_ (Brown, 2010).

## 3 CHOLINERGIC NEURONS IN THE SPINAL CORD

**FIGURE 1:**
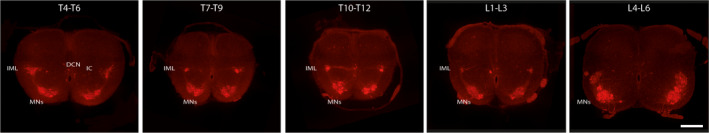
Locations of spinal cholinergic neurons. Spinal cord immunofluorescence labeling for choline acetyltransferase (ChAT, lighter red staining) at different indicated thoraco (T)‐lumbar (L) levels. IML: intermediolateral column, MNs: motoneurons, DCN: dorsal commissural nucleus, IC: intercalated nucleus. Calibration bar: 100 μm

The anatomical identification of spinal neurons that use ACh as a neurotransmitter was originally achieved by histological or immunohistological detection of the different proteins involved in ACh metabolism (ChAT, VAChT, and AChE; Anglister et al., 2008; Arvidsson et al., 1997; Barber et al., 1984; Li et al., 1995). More recently, reporter transgenic mice expressing enhanced green fluorescent protein under the control of the ChAT promoter (ChAT‐eGFP mice) have enabled the direct visualization of spinal cholinergic neurons (Tallini et al., 2006). The most abundant class of spinal cholinergic cells comprises somatic MNs located ventrally and extending from the cervical to sacral cord segments (Figure 1). In the spinal cord of mammals, small cholinergic neurons scattered around the central canal (lamina X) and in the superficial laminae of the dorsal horn are also found in all spinal segments, as well as large cell body neurons located at the border between the dorsal and ventral part of the cord in lamina VII (the partition cells). During locomotion, it has been shown that in addition to MNs, both partition cells and small interneurons of lamina X are active in the L3‐S1 segments of the cat spinal cord (Huang et al., 2000).

In the mammalian spinal thoracolumbar and sacral segments, these four types of cholinergic neurons are intermingled with cholinergic preganglionic neurons of the autonomic nervous system (ANS) that send their axon in the ventral roots of the spinal cord to synapse with postganglionic neurons. While parasympathetic preganglionic neurons are located in the brainstem and sacral segments, the sympathetic preganglionic neurons (SPNs) span from the first thoracic (T1) to the second or third lumbar segments (L2–L3) in rodents and humans (Barber et al., 1984; Deuchars & Lall, 2015; Krassioukov et al., 1998). Figure 1 shows the three different subpopulations of SPNs distributed in the intermediolateral nucleus (IML SPNs), the intercalated nucleus (IN), and in the region above the central canal (the dorsal commissural nucleus, DCN) in thoracic segments, but not in the caudal lumbar segments.

## 4 CHOLINERGIC NEUROMODULATORY EFFECTS ON SPINAL LOCOMOTOR NETWORKS

As previously mentioned, the source of ACh in the spinal cord appears to be purely intraspinal. None of the descending pathways originating from supraspinal structures that project to the spinal cord have been shown to be cholinergic (Rye et al., 1988; Sherriff et al., 1991; VanderHorst & Ulfhake, 2006). Moreover, spinal cholinergic synapses and their associated receptors have been found to be relatively preserved after a spinal cord injury (SCI), further pointing to their spinal origin (Kanazawa et al., 1979; Kayaalp & Neff, 1980; Vulovic et al., 2018). Although cholinergic neurons are present in all spinal segments, there is no direct evidence as to whether cholinergic modulation occurs at the segmental level on spinal locomotor neurons. In contrast, cholinergic intersegmental interactions have been identified. Cholinergic neurons that connect monosynaptically with MNs innervating limb quadriceps muscle have been found throughout the T11‐L6 spinal segments (Stepien et al., 2010). Moreover, ascending cholinergic inputs arising from the sacral and thoracolumbar part of the cord have also been reported (Anglister et al., 2017; Carlin et al., 2006; Finkel et al., 2014).

### 4.1 Nicotinic effects in locomotor spinal networks

In the spinal cord, nAChRs are highly expressed, especially in the dorsal part where they control the integration of innocuous and nociceptive inputs (for review see Naser & Kuner, 2018). In the ventral spinal cord, MNs have been shown to be depolarized by nAChR activation in numerous animal models (Blake et al., 1987; Ogier et al., 2004; Perrins & Roberts, 1994; Quinlan & Buchanan, 2008). In addition to their direct excitatory effects on MNs, nAChRs are involved in a very particular function in spinal motor networks, namely recurrent inhibitory feedback onto MNs. For this, MN axons send local collateral branches to synapse with glycinergic neurons, the Renshaw cells, which in turn synapse back onto MNs. When MNs are active, the release of ACh (as well as the co‐release of glutamate (Mentis et al., 2005)) and the subsequent activation of specific subtypes of nAChRs leads to Renshaw cell activation, which then inhibits the MNs (Alvarez & Fyffe, 2007; Bhumbra & Beato, 2018; Lamotte d'Incamps et al., 2018). While the precise role of this recurrent inhibitory circuit is still under debate, it undoubtedly serves as a powerful mechanism for controlling MN excitability.

### 4.2 Muscarinic effects in spinal locomotor networks

A large part of the neuromodulatory processes occurring in spinal circuits at both dorsal and ventral levels results from mAChR activation (Davis, 2013; Miles & Sillar, 2011). Among the five mAChR subtypes, the presence of the M2, M3, M4, and M5 receptors in the spinal cord has been established by anatomical and biochemical studies (see, for examples: Höglund & Baghdoyan, 1997; Wei et al., 1994; Yung & Lo, 1997). The spinal expression of M1 receptors has also been observed, but only on the basis of pharmacological experiments using M1 antagonists (see for examples: Bertrand & Cazalets, 2011; Naguib & Yaksh, 1997; Zhuo & Gebhart, 1991).

In isolated spinal cord preparations from newborn rodents, it is possible to elicit by pharmacological or electrical stimulations a rhythmic bursting activity that shares many features with the locomotor pattern recorded in vivo (Cazalets et al., 1992; Kudo & Yamada, 1987; Pujala et al., 2016; Magnuson & Trinder, 1997). This activity produced in the absence of muscles and sensory inputs was termed “fictive locomotion” and consists of right‐left and homolateral flexor‐extensor alternating bursts of action potentials recorded from the lumbar ventral roots. When edrophonium (EDRO), an AChE inhibitor, is applied to the in vitro spinal cord preparation of newborn rodents, alone or in combination with ACh, it often induces a slow non locomotor‐like activity characterized by left/right alternating motor bursting, but an absence of homolateral flexor/extensor alternation (Anglister et al., 2008; Cowley & Schmidt, 1994; Kiehn et al., 1996). A recent study reexamined this AChE inhibitor‐induced activity and revealed that within the slow nonalternating flexor/extensor bursts of activity, a well‐coordinated fictive locomotory pattern could be observed (Jordan et al., 2014). The EDRO‐induced rhythm is insensitive to nAChR antagonists but is abolished in the presence of mAChR antagonists such as atropine (Jordan et al., 2014; Smith et al., 1988) and appears to require the synergistic activation of M3 receptors on locomotor CPG interneurons and M2 receptors on MNs (Jordan et al., 2014). Moreover, slow nonlocomotor rhythms have been observed in the presence of oxotremorine, a nonselective mAChR agonist, in the in vitro spinal cord preparation of newborn rodents (Sourioux et al., 2018) and turtles (Guertin & Hounsgaard, 1999). AChE blockers, nonselective mAChR agonists and antagonists, as well as selective M2 receptor antagonists have been shown to modulate ongoing fictive locomotion expressed spontaneously or induced with noncholinergic neuroactive compounds (Bertrand & Cazalets, 2011; Fok & Stein, 2002; Milan et al., 2015; Miles et al., 2007; Nascimento et al., 2019; Perrins & Roberts, 1995; Quinlan et al., 2004).

A substantial research literature describing the various cellular targets of the muscarinic cholinergic system in spinal locomotor networks is now available. mAchR activation has been shown to strongly depress the sensory afferent inputs received by MNs (Chen et al., 2014; Zhang et al., 2007). It has been reported that muscarinic agonists decrease glutamatergic synaptic transmission onto MNs (Kurihara et al., 1993) through M2 receptor activation, whereas M3 receptor activation tends to increase transmission (Nascimento et al., 2019). A muscarinic‐dependent short‐term potentiation of commissural glutamatergic transmission has also been described onto MNs (Bertrand & Cazalets, 2011). In rodents, turtles, and salamanders, mAChR activation has been shown to mediate a global increase in MN excitability (Chevallier et al., 2006; Ghezzi et al., 2017; Ireland et al., 2012; Miles et al., 2007) by: (1) inhibiting several types of potassium currents (such as the M3 receptor‐associated noninactivating K^+^ current, the M‐current (Alaburda et al., 2002; Bertrand & Cazalets, 2011; Ghezzi et al., 2017), the inwardly rectifying K^+^ current (Chevallier et al., 2006)), the delayed rectifier K^+^ current (Nascimento et al., 2020), as well as the hyperpolarization‐activated cation current I_H_ (Chevallier et al., 2006; Ireland et al., 2012); (2) inducing plateau‐like membrane depolarizations (Alaburda et al., 2002; Svirskis & Hounsgaard, 1998), (3) reducing after‐spike hyperpolarization (AHP; Chevallier et al., (2006)) and the related Ca^2+^‐activated K^+^ current through M2 receptor activation (Miles et al., 2007); and (4) activating voltage‐gated Ca^2+^ channels (Svirskis & Hounsgaard, 1998). It should be noted that in both lampreys and *Xenopus* tadpoles, muscarinic agonists have little effect or only trigger a slight hyperpolarization of MNs (Perrins & Roberts, 1994; Quinlan & Buchanan, 2008). In addition to species differences, recent data have suggested that the muscarinic modulation of MN excitability could also differ according to MN subtype. In the zebrafish, muscarine increases the firing rate of slow and intermediate secondary axial MNs but decreases the excitability of fast MNs. Such differential effects have been shown to rely at least partly on differential mAChR expression between MN subtypes (Bertuzzi et al., 2018). In the mouse in vitro spinal cord preparation, muscarine has been reported to trigger a M2 receptor‐dependent outward current in small MNs, but a M3 receptor‐dependent inward current in large MNs (Nascimento et al., 2019).

The majority of the muscarinic modulatory effects described above imply the involvement of M2 and M3 mAChRs. Although the M1, M4, and M5 receptor subtypes have been shown to be present in the spinal cord, their role is still largely overlooked. Using selective mAChR antagonists in the isolated spinal cord of the newborn rat, our group has shown that the M1 and M4 receptors participate in oxotremorine‐induced depolarization of lumbar MNs, and that M4 receptors contribute to the cholinergic response to a commissural stimulation (Bertrand & Cazalets, 2011).

### 4.3 Somato‐sympathetic coupling: A cholinergic origin

**FIGURE 2:**
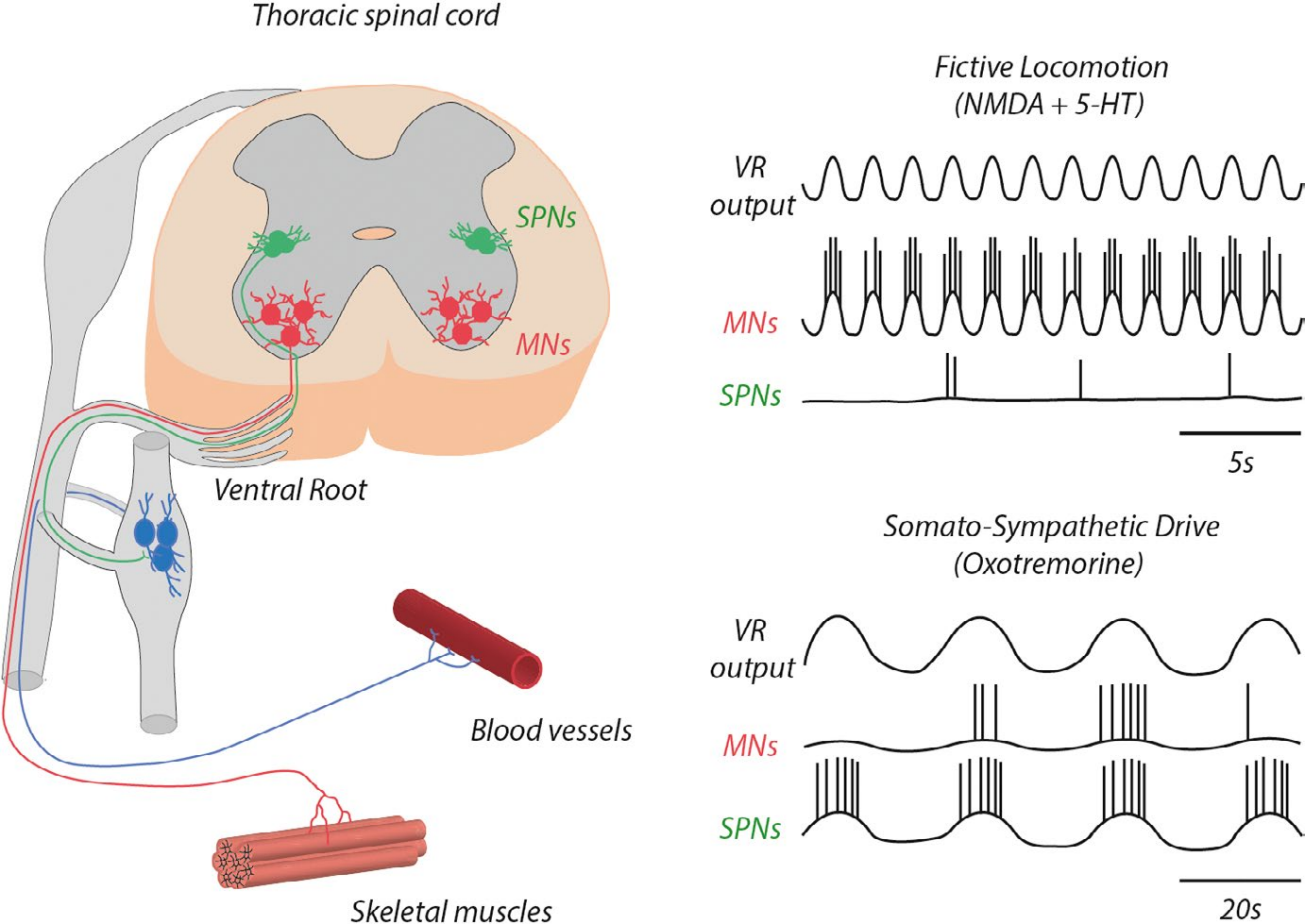
Somato‐sympathetic coupling. The specific activation of muscarinic cholinergic receptors (mAChRs) with oxotremorine triggers a slow burst rhythm in thoracic spinal segments. Whereas motoneurons (MNs) are rhythmically active during both fictive locomotion induced by NMDA and serotonin (5‐HT) and the oxotremorine‐induced slow bursting, intermediolateral sympathetic preganglionic neurons (SPNs) express rhythmicity solely in the presence of oxotremorine. This common synaptic influence shared by MNs and SPNs has been called the somato‐sympathetic drive

As previously mentioned, AChE inhibitors and mAChR agonists trigger rhythmic activity in the lumbar segments of the spinal cord. We have recently shown that the exogenous application of oxotremorine on isolated thoracic segments induces the expression of a slow motor rhythm in thoracic ventral roots (Sourioux et al., 2018). We observed that during this oxotremorine‐induced rhythm, both thoracic MNs and SPNs located in the thoracic segments are rhythmically active (Figure 2). In contrast, when fictive locomotion is induced by NMDA receptor activation (with exogenous NMDA) in combination with serotonin (5‐hydroxytryptamine, 5‐HT), only the MNs are rhythmically active (Figure 2). In addition to revealing for the first time a distinct rhythmogenic capability of the thoracic cord region, these findings were able to shed light on a functional intraspinal coupling between the somatic and sympathetic systems, the triggering mechanism for which is dependent on mAChR activation (Sourioux et al., 2018). We hypothesize that the somato‐sympathetic drive we described could be the basis of the periodic blood pressure variations observed at rest with a period of approximately 10 s, the Mayer waves (Julien et al., 2001).

### 4.4 Cholinergic synapses and C boutons

To the best of our knowledge, there is so far no direct evidence for the presence of cholinergic synapses on the distal dendritic tree of MNs, although they almost certainly exist. In contrast, a very easily accessible and particular type of cholinergic synapse located on MN somata and proximal dendrites is very well documented: the C boutons (for reviews see: Deardorff et al., 2014; Witts et al., 2014). Described for the first time in cat MNs (Conradi, 1969), C boutons are large (2–7 μm) synaptic contacts containing spherical synaptic vesicles associated with a highly specialized postsynaptic structure, the subsurface cisternae (SSC) that gave its name to the synapse (Conradi et al., 1979). C boutons seem to be a hallmark of alpha MNs, since gamma MNs, spinal interneurons, and Renshaw cells are devoid of this type of synapse (Deardorff et al., 2014; Hellström et al., 2003). C bouton synapses impinging on MNs were shown to derive from V0c cholinergic interneurons identifiable by their expression of the V0‐specific homeobox protein Dbx1 and the paired‐like homeodomain transcription factor Pitx2, and are located in lamina X and the most medial part of lamina VII (Miles et al., 2007; Zagoraiou et al., 2009). Numerous studies have deciphered the cytoarchitecture of C boutons and described the different proteins present in the pre‐ and postsynaptic compartments, such as M2 mAChRs (Hellström et al., 2003), SK2/3 subtypes of Ca^2+^‐dependent K^+^ channels (Brownstone & Magown, 2013; Deardorff et al., 2013) as well as voltage‐activated Kv2.1 channels (Muennich & Fyffe, 2004) at the postsynaptic level and N‐type voltage‐activated Ca^2+^ channels (Cav2.2; Wilson et al., 2004) in the C bouton terminals facing MNs. The activation of M2 mAChRs in C boutons has been proposed to block directly or indirectly (via N‐type Ca^2+^ channel inhibition) the SK channel and shorten spike duration through Kv2.1 delayed rectifier channel activity, leading to a decrease in AHP amplitude and an increase in MN excitability (Deardorff et al., 2014; Miles et al., 2007; Nascimento et al., 2019). Very recently, a new postsynaptic partner of C boutons has been characterized: TMEM16F, a newly emerging family of calcium‐activated chloride channels (Soulard et al., 2020). Furthermore, sigma 1 (S1R) (Mavlyutov et al., 2010) and neuroligin 1 (NRG1) receptors (Gallart Palau et al., 2014) have been found at the SSC surface in the postsynaptic C bouton complex. SSC are intracellular Ca^2+^ stores from which Ca^2+^ release is controlled by the S1R (Kourrich et al., 2012), and in turn contributes to the activation of SK channels and AHP generation. Finally, nAChRs and P2X7 ATP receptors were found in the presynaptic compartment of the C bouton and may contribute to the postsynaptic regulation of ACh release (Deng & Fyffe, 2004; Khan et al., 2003). Several studies have provided data on the functional implication of C boutons. The impact of the functional silencing of these cholinergic synapses has been assessed at the behavioral level in a mouse transgenic line in which ChAT synthesis in V0_C_ neurons is specifically suppressed. These animals exhibit a preserved locomotor pattern, but exhibit impairments in motor task‐dependent modulation, suggesting a crucial role of C boutons in MN recruitment and modulation (Zagoraiou et al., 2009). More recently, Nascimento et al., (2020) showed that the chemogenetic excitation of Pitx2 neurons leads to an increase in motor output through the activation of M2 mAchRs and Kv2.1 channels at C boutons. This study is in agreement with the demonstration of the salient role of C bouton‐associated Kv2.1 channels in the regulation of MN firing rate (Romer et al., 2014). In the zebrafish spinal cord, the activation of mAchRs has been shown to alter the operational range of locomotor networks by both facilitating the recruitment of slow and intermediate MNs and depressing fast MN excitability (Bertuzzi & Ampatzis, 2018). Altogether these data reveal that the spinal cholinergic system plays a major role in the state‐dependent modulation of motor behaviors.

## 5 THE CHOLINERGIC SPINAL SYSTEM IN PATHOPHYSIOLOGICAL CONDITIONS

Altogether, the data gathered on the propriospinal cholinergic system show that it acts through two different types of receptors on all neuronal elements of spinal motor circuitry and plays a major role in network function. Consequently, due to this central role, defects in cholinergic neuromodulatory processes have been consistently associated with spinal motor disorders.

### 5.1 Amyotrophic lateral sclerosis (ALS) and C boutons

ALS is a fatal neurodegenerative disorder characterized by the selective degeneration of cortical motor neurons and spinal MNs. Excitability deregulation of MNs has been reported as an important factor in ALS (Jensen et al., 2020; Leroy et al., 2014; Martin et al., 2013; Martínez‐Silva et al., 2018) and C bouton synaptic terminal coverage has been shown to differ between MN subtypes, being more prevalent in the most vulnerable fast MNs in ALS (Hellström et al., 2003). Accordingly, numerous studies have been conducted to assess whether an alteration in C bouton morphology occurs during ALS pathogenesis (Casas et al., 2013; Dukkipati et al., 2017; Herron & Miles, 2012; Milan et al., 2015; Pullen & Athanasiou, 2009; Saxena et al., 2013). Although providing contradictory results, these studies have all demonstrated impairments of C bouton cytoarchitecture during the ALS pathogenesis (Casas et al., 2013; Dukkipati et al., 2017; Herron & Miles, 2012; Milan et al., 2015; Pullen & Athanasiou, 2009; Saxena et al., 2013). Moreover, we showed that MNs are not the only spinal neurons to degenerate during ALS, but that cholinergic neurons located in lamina X also undergo cell death processes during the disease's progression (Milan et al., 2015). The physiological consequences of these alterations are still largely unknown, and work is currently in progress to understand further the precise cholinergic inputs received by the different MN subtypes and whether they are at least partly responsible for the fast MNs’ selective vulnerability in ALS. A recent study reported that the genetic inactivation of C boutons in the superoxide dismutase 1 (SOD1) mouse model of ALS improves motor performances in early symptomatic stages of ALS, but fails to promote mouse survival (Konsolaki et al., 2020).

### 5.2 The cholinergic spinal system after spinal cord injury (SCI)

Lesions of the spinal cord tend to isolate spinal networks from the brain's influence by disconnecting descending pathways from their spinal targets. It is well known that both MNs and SPNs receive a variety of supraspinal synaptic commands (Deuchars & Lall, 2015; Rekling et al., 2000), although there is no direct evidence for supraspinal originating inputs impinging on propriospinal cholinergic interneurons. Pitx2‐expressing neurons have been shown to connect ipsilateral MNs and to be driven by local interneurons during fictive locomotion, but their other synaptic partners are so far unknown. (Zagoraiou et al., 2009). However, following SCI, the spinal cholinergic system has been shown to undergo substantial remodeling (Kanazawa et al., 1979; Kayaalp & Neff, 1980; Vulovic et al., 2018). A unilateral lesion of the corticospinal tract in rats causes a massive elimination of ChAT‐positive neurons in lamina X and associated C boutons in the cervical segments, through microglial phagocytosis (Jiang et al., 2018). Additionally, it has been shown that genes coding for nAChR subunits and Ach‐releasing machinery are upregulated following a complete sacral SCI, which could lead to an increase in both ACh sensitivity and release (Wienecke et al., 2010). These data are consistent with the observation that the spinal networks exhibit a hypercholinergic state following SCI (Jordan et al., 2014). The contribution of propriospinal cholinergic neurons to functional recovery after SCI is still unclear, although high levels of ChAT immunofluorescence were shown to be strongly correlated with motor recovery after an incomplete SCI in rats (Nakamura et al., 1996). In contrast, in spinal cat and rat, the activation of the spinal cholinergic system appears to be detrimental to locomotor expression, whereas it is improved with cholinergic antagonist administration (Jordan et al., 2014). These authors hypothesized that a strong cholinergic inhibition of sensory afferent inputs could explain their results and suggested that cholinergic antagonists could serve as potent therapeutic drugs to promote motor recovery after SCI.

## 6 CONCLUSIONS

The cellular basis of cholinergic neuromodulatory processes in spinal locomotor networks appears to be highly complex and the recent discovery of novel molecular targets, such as TEM16F, suggests that we are far from understanding the full range of this system's actions. We now have some insights into the role of Pitx2 neurons, but important unanswered questions remain: what are the functions of the other propriospinal cholinergic neurons such as non‐Pitx2 V0c neurons, partition cells…, do SPNs participate in the cholinergic neuromodulation of spinal locomotor networks, and what are the presynaptic partners of these different cell types? The unique position of the cholinergic neurons as a purely propriospinal neuromodulatory system makes it a prime target for normalizing or reactivating locomotor functions in pathophysiological conditions.

## CONFLICT OF INTEREST

The authors have no conflict of interest.

## AUTHOR CONTRIBUTIONS

TM, CG, JRC, and SSB wrote and revised the manuscript.
